# TRPC4/5 inhibitors: Phase I results and proof of concept studies

**DOI:** 10.1007/s00406-024-01890-0

**Published:** 2024-09-29

**Authors:** Simone Grimm, Stefan Just, Rene Fuertig, Jennifer B. Dwyer, Vikas M. Sharma, Andreas Wunder

**Affiliations:** 1 Medical School Berlin, Rüdesheimer Str., 5014197 Berlin, Germany; 2 Department of Psychiatry, Campus Benjamin Franklin Charité, Berlin, Germany; 3 Boehringer Ingelheim Pharma GmbH & Co. KG, Biberach an der Riss, Germany; 4 Boehringer Ingelheim Pharmaceuticals, Ridgefield, CT USA; 5 Boehringer Ingelheim International GmbH, Ingelheim am Rhein, Germany

**Keywords:** Transient receptor potential canonical ion channels, Negative valence systems, Emotional processing, Major depressive disorder, Anxiety, Neuroimaging

## Abstract

Transient receptor potential canonical (TRPC) ion channels are expressed in areas of the brain responsible for processing emotion and mood and have been implicated in the pathophysiology of internalizing disorders such as major depressive disorder and anxiety disorders. This review outlines the rationale for targeting TRPC ion channels for drug development, with specific focus on TRPC4 and TRPC5. We provide preclinical evidence that the lack of TRPC4 and TRPC5 channels or its pharmacological inhibition attenuate fear and anxiety without impairing other behaviors in mice. We also report on clinical studies of BI 1358894, a small molecule inhibitor of TRPC4/5 ion channels, demonstrating reduced psychological and physiological responses to induced anxiety/panic-like symptoms in healthy volunteers. Furthermore, we highlight an imaging study that investigated the acute effects of BI 1358894 and showed reduced activation in several brain regions involved in emotional processing. We conclude that these findings demonstrate a critical role for TRPC4 and TRPC5 in emotional processing, even though it remains an open question if the biological signatures of TRPC4/5 inhibition reported here translate into clinical efficacy and indicate that a TRPC4/5 inhibitor might provide a more effective treatment of internalizing disorders.

## Introduction

Internalizing disorders, such as major depressive disorder (MDD) and anxiety disorders pose a significant public health threat and are one of the leading causes of disability worldwide with numbers still rising [1]. Furthermore, anxiety and comorbid anxiety disorders are highly prevalent in individuals with MDD, with occurrence of lifetime anxiety disorders in MDD patients estimated at 46–78% [2, 3]. Even in the absence of a formal anxiety disorder, high levels of anxiety are common in people with MDD [4]. Although treatment with antidepressants is often effective, a significant number of patients do not respond adequately to established treatments [5, 6], and it is not yet possible to predict which treatment will be effective for a given patient [7]. Given the unsatisfactory efficacy of traditional antidepressants [8–10] and the limited number of patients who respond sufficiently to them [6], the search for more effective treatments has become an imperative for psychiatry.

Rather than the trial-and-error approach to treatment selection [11], neuroimaging technologies could facilitate treatment development by identifying objective neurobiological markers underlying pathophysiological processes in mental disorders [12]. In this sense, rather than defining disorders in terms of nosological categories based on clusters of symptoms, the Research Domain Criteria (RDoC) paradigm [13] provides a framework that takes into account neurobiological evidence and is based on the assumption that mental disorders are associated with dysfunctional brain circuits that can be identified neuroscientifically (for example, with functional neuroimaging). Neuroimaging could therefore contribute to the development of biosignatures (“constructs”) that underlie core symptoms [14, 15]. Among the five major domains within the RDoC framework, negative valence (NV) systems, which control responses to aversive stimuli, have been linked to core symptoms of internalizing disorders such as dysphoria and anxiety, and may therefore be crucial to understanding them [16]. NV systems involve corticolimbic regions such as the amygdala, insula, dorsolateral prefrontal cortex (DLPFC) and anterior cingulate cortex (ACC) [16, 17]. These regions are all part of the neural network relevant for the processing of emotions [18]. Abnormal emotional processing with preferential processing of negative information has been consistently demonstrated in MDD. Particularly, depressed individuals are more likely to evaluate their environment according to negative schemas and show increased memory and attention to negative stimuli [19]. This mood-congruent affective bias has been associated with hyperactivity to negative stimuli in the amygdala [17, 20], a brain region crucial for identifying the emotional significance of a stimulus and generating emotional responses. Recent evidence also suggests that amygdala hyperactivity in response to emotional faces may be a neurobiological feature specific to MDD with anxious distress compared with MDD without anxious distress [21]. MDD is also characterized by maladaptive emotion regulation strategies and difficulties in regulating negative emotions, which might be related to deficits in executive control as mediated by DLPFC and ACC activity [22]. Several functional magnetic resonance imaging (fMRI) studies in healthy volunteers report an acute effect of antidepressants on brain regions implicated in emotion processing [23–26]. However, if, how, and how quickly this translates to clinical efficacy in internalizing disorders remains unclear. In MDD, longer-term (i.e., weeks) administration of antidepressants does however affect emotional circuitry by decreasing activity to negative emotions in the amygdala, insula, and ACC, and by increasing regulatory responses in the DLPFC. Thus, one might speculate that through these mechanisms, antidepressants may eventually improve depressed patients’ abnormalities in emotional reactivity and deficits in emotional regulation [20].

Similarly, in individuals with post-traumatic stress disorder (PTSD), morphological and functional changes in the amygdala are evident, resulting in disrupted control of fear generalization, arousal, and reward processing. Specifically, decreased amygdala nuclei volumes are reported to be associated with symptoms of PTSD [27, 28]. Furthermore, reduced grey matter volume in limbic brain regions including the amygdala are correlated with sleep disturbances (insomnia and nightmares) in patients with PTSD which may contribute to symptom severity. In line with RDoC framework and considering the evidence implicating amygdala involvement in PTSD symptoms, the location of both TRPC4 and TRPC5 in the amygdala and the reduced fear and anxiety demonstrated in TRPC4^−/−^ and TRPC5^−/−^ knockout mice [29, 30], it is reasonable to hypothesize that dampening amygdala activation of the fear response by blocking these ion channels may reduce PTSD symptom severity [31].

## TRPC4/5 as a target for drug development

Transient receptor potential canonical (TRPC) ion channels are an attractive potential therapeutic target for treating MDD and PTSD. Firstly, they are expressed in areas of the rodent brain responsible for processing emotion and mood [32]. Secondly, interactions between TRPC ion channels and neurotransmitter networks have been implicated in the pathophysiology of MDD [33].

## Biology of TRPC4/5

**Fig. 1:**
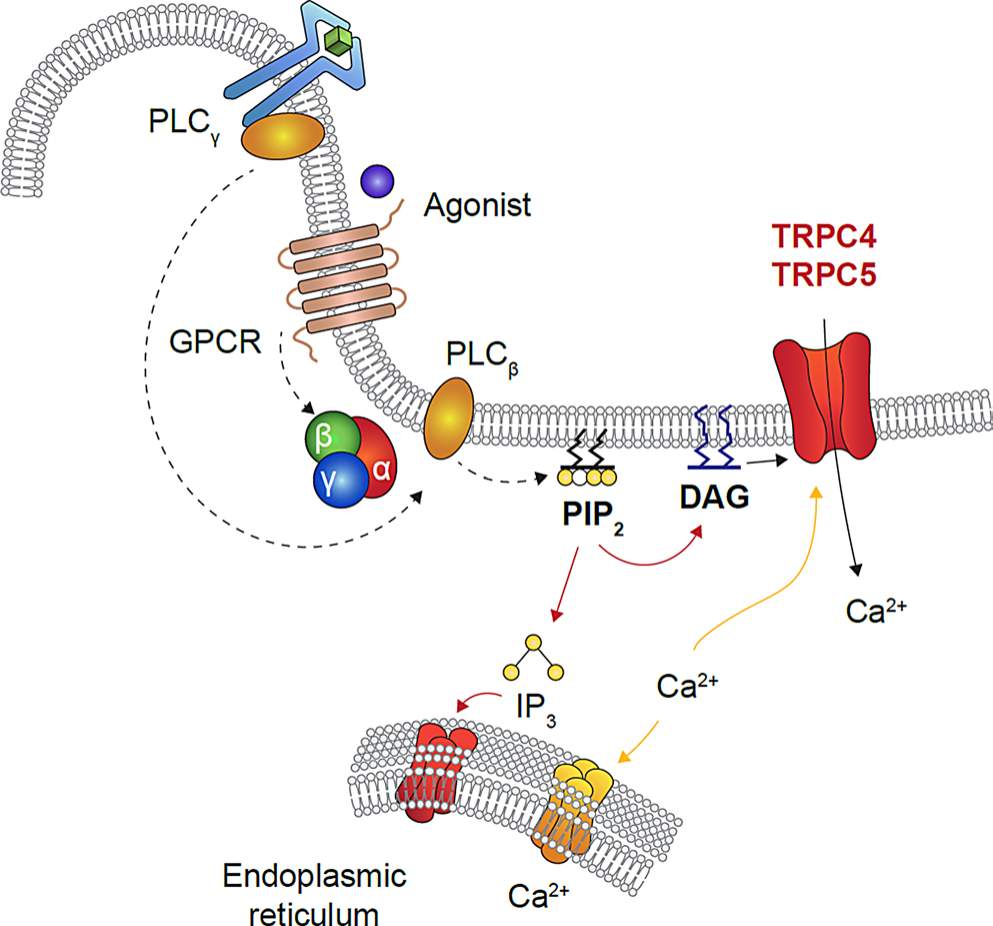
**GPCR activation and regulation of TRPC channels.** Binding of an agonist to GPCR initiates a signaling cascade, stimulation of G protein causes phospholipase C-mediated hydrolysis of PIP_2_ to IP_3_ and diacylglycerol (DAG). IP_3_ binds to IP_3_ receptor, a ligand-gated ion channel on the endoplasmic reticulum (ER), which leads to the release of Ca^2+^ from the internal ER stores. This Ca^2+^ depletion in turn, allows stromal interaction molecule 1 (STIM1) to aggregate, followed by the activation of the TRPC channels in the plasma membrane, allowing Ca^2+^ to enter the cell that orchestrates cellular functions [85, 86]. DAG, diacylglycerol; GPCR, G protein coupled receptor; IP_3 ,_ inositol-triphosphate; PIP_2_, bisphosphate; PLC, phospholipase C; TRPC, transient receptor potential canonical. Adapted from Selvaraj S, et al. *CNS Neurol Disord Drug Targets*. 2010;9:94–104. 10.2174/18715271079096665.

TRPC4 and TRPC5 are members of a superfamily of transient receptor potential (TRP) channels that consists of 28 nonselective cation channels with varied physiological functions [34, 35]. The TRPC subfamily consists of seven members, which are organized into four groups based on sequence homology and functional similarities: TRPC1, TRPC2, TRPC4/5, and TRPC3/6/7, although some suggest TRPC1 should be grouped with TRPC4/5 [34, 36–38]. TRPC4 and TRPC5 can be modulated by a range of physiological factors, and physical and chemical stimuli, such as temperature, redox status, endogenous and dietary lipids, heavy metal ions, and synthetic small molecules [39]. Both TRPC4 and TRPC5 can be potentiated by G protein-coupled receptors (GPCRs) that couple to G-alpha q/11 and/or G-alpha i/o and receptor tyrosine kinases, which activate phospholipase C. In turn, this leads to an increase in inositol triphosphate (IP_3_) that binds to the IP_3_ receptor on the endoplasmic reticulum [40–44]. This cascade signals the release of intracellular calcium or vesicular translocation to the membrane, current induction, and physiological responses [43, 45–48] (Fig. 1).

The role of TRPC1/4/5 in anxiety, depression, and fear-related behavior [29, 49], is a widely researched area, supported by TRPC4 and TRPC5 expression in the brain and by evidence from both TRPC modulators and transgenic mouse models [29, 30]. TRPC4 and TRPC5 are widely expressed in the brain, predominantly in areas of the corticolimbic system that regulate emotional processing and stress, including the prefrontal cortex, orbitofrontal cortex, lateral septum, pyramidal cell layer of the hippocampus, and amygdala [32, 50, 51]. Murine models have implicated TRPC4 and TRPC5 activation in fear-related behaviors and anxiety [29, 30]. Activation of G-alpha q coupled-GPCRs of the amygdala were associated with fear-related behaviors in a mouse behavior paradigm of fear-potentiated startle [52]. Furthermore, genetic deletion of either TRPC4 or TRPC5 can reduce anxiety behaviors in mice [29, 30]. In the open field task, which assesses anxiety-like behavior arising from the conflict between exploring novel areas and a desire to actively avoid brightly lit, open areas; mice lacking either TRPC4 or TRPC5 showed increased exploratory behavior compared with wild-type litter mates, spending more time in the anxiogenic center of the arena and entering the center more frequently than wild-type mice [29, 30]. Similarly, in elevated plus maze tests, mice lacking TRPC4 and TRPC5 were less anxious than their wild-type litter mates, being more likely to enter the open arms of the maze and more likely to stay there [29, 30]. Further studies in TRPC4 and TRPC5 null mice indicate that deficits in fear-related behavior may be due to the lack of TRPC activation resulting in diminished Group I mGluR-mediated synaptic responses and cholecystokinin 2 (CCK-2) receptor-activated signaling of neurons in the lateral nucleus of the amygdala [29, 30]. Furthermore, targeted knockdown of TRPC4 in the amygdala of mice using RNA interference produced an anxiolytic phenotype similar to TRPC4 null mice, further implicating TRPC4 in the amygdala in the control of innate fear [30]. Collectively, these results suggest that TRPC4/5 inhibition may offer a potential neuronal signaling mechanism that could be targeted therapeutically for the treatment of anxiety and depression.

## Anxiolytic and antidepressant effects of TRPC4/5 inhibition in preclinical studies

Depression, anxiety, and panic disorder are often comorbid and may be mediated through the same neuronal networks [53–55]. CCK-tetrapeptide (CCK-4) is a synthetic analog of the endogenous neuropeptide CCK [56]. CCK-4 can induce anxiety/panic-like symptoms when administered to humans and is believed to be implicated in the neuronal network of panic disorder [56]. The CCK-4 challenge model is a well-accepted and applied model for investigating the pathophysiology of anxiety/panic in animals as well as in humans [54, 56–58].

**Fig. 2:**
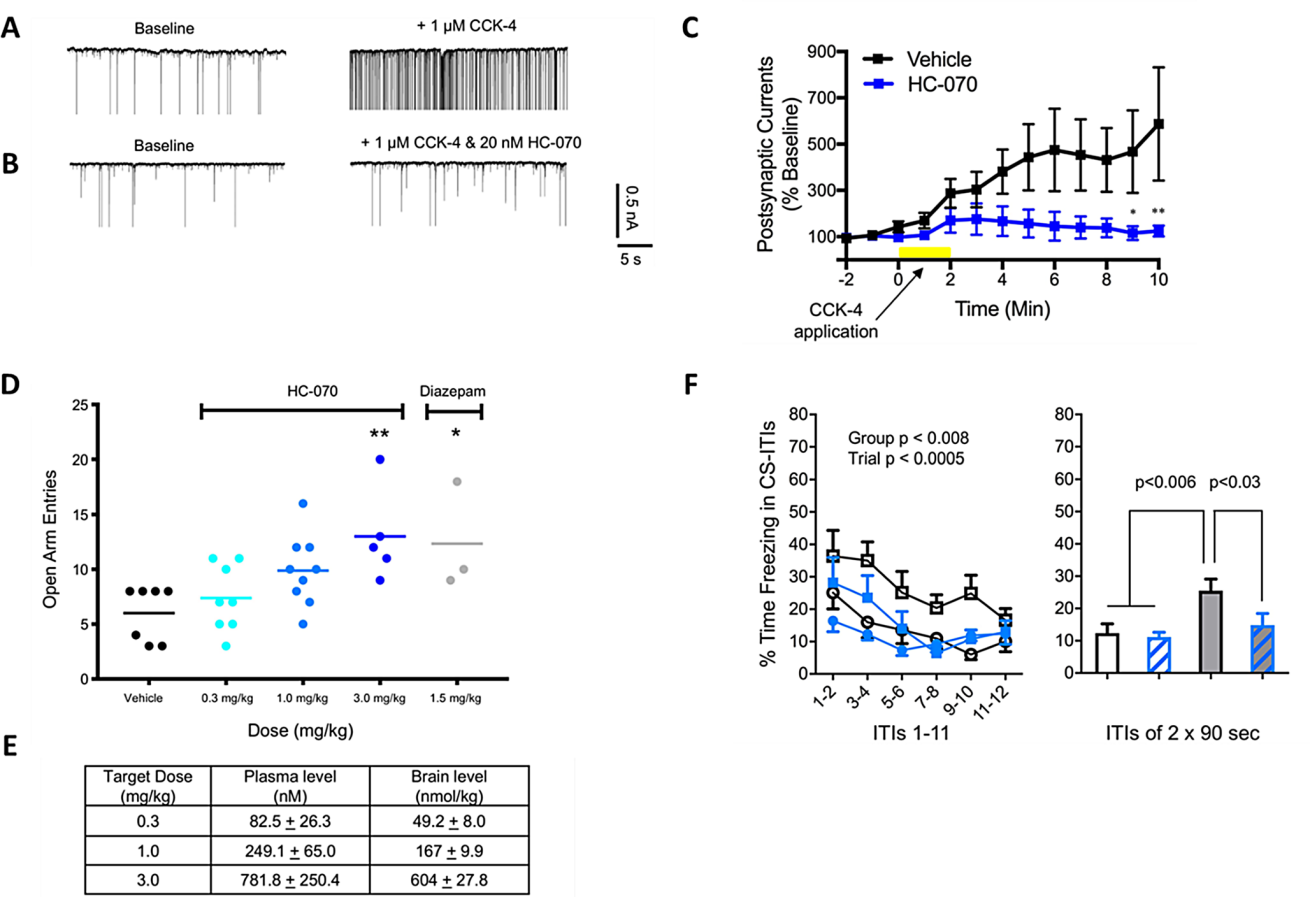
**Preclinical data in mouse models indicates that the TRPC4/5 inhibitor HC-070 has anxiolytic and anti-depressant effects. (A–C) HC-070 attenuates CCK-4-induced EPSCs recorded from the basolateral amygdala in a slice preparation**. (**A**) Representative traces before and after CCK-4 application in the presence of vehicle. (**B**) Representative traces before and after CCK-4 application in the presence or absence of HC-070. (**C**) Quantitation of the results (*n* = 8). HC-070 was pre-incubated with the slice. CCK-4 was applied at the time indicated. Error bars represent the SEM (*p < 0.05, **p < 0.001, Tukey’s multiple comparisons test following two-way analysis of variance [ANOVA]). The holding potential was − 70 mV. **(D & E) HC-070 decreases anxiety in a standard EPM**. (**D**) Mice were administered vehicle or 0.3, 1, or 3 mg/kg HC-070 orally 60 min prior to EPM. The positive control, 1.5 mg/kg diazepam, was administered IP 30 min prior to testing. At 3 mg/kg, HC-070 significantly increased the number of open arm entries compared to the oral control (**p < 0.01, Dunnett’s post-hoc test following one-way ANOVA). The positive control, 1.5 mg/kg diazepam, also increased open arm entries (*p < 0.05, t-test). Animals that jumped or fell off the EPM during the test were excluded, such that the sample size (n) differed between groups. Each animal is shown on the graph (circles) and the horizontal lines represent the mean. **(E)** Average plasma and brain exposures from satellite animals dosed with 0.3, 1, or 3 mg/kg HC-070, 60 min post-dosing. Error bars show standard deviation. **(F) Effects of HC-070 on CSD-induced fear hyper-reactivity.** In the tone CS memory test, freezing was also measured in the inter-trial intervals (ITIs) between CS presentations. The left panel presents the fear expression curve in ITIs between CSs using the average freezing per pair of consecutive ITIs. The right panel presents the average freezing across all 12 ITIs. Here also, HC-070 reduced fear memory in CSD mice, as indicated by the increased rate at which their freezing level during the ITIs attenuated compared with CSD-VEH mice, i.e., faster extinction learning. ANOVA, analysis of variance; CCK-4, cholecystokinin-tetrapeptide; CS, conditioned stimulus; CSD, chronic social defeat; EPM, elevated plus maze; EPSC, excitatory postsynaptic current; ITI, inter-trial interval; SEM, standard error of the mean; TRPC, transient receptor potential conical; VEH, vehicle. Reproduced from Just S, et al. *PLoS One.* 2018;13(1):e0191225 under the terms of the Creative Commons Attribution License (http://creativecommons.org/licenses/by/4.0/).

HC-070 is a highly potent, small molecule antagonist of TRPC4/5 [59]. In preclinical studies, HC-070 20 nM attenuated CCK-4-induced excitatory postsynaptic current frequency, recorded from the basolateral amygdala in a slice preparation (Fig. 2A–C) [59]. Additionally, during elevated plus maze tests, treatment with HC-070 led to a dose-dependent reduction in CCK-induced anxiety-like behavior compared with vehicle control, with a magnitude of reduction similar to that observed in mice treated with the anxiolytic, diazepam (Fig. 2D and E) [59]. In a model of chronic social defeat, which assesses the impact of prolonged exposure to social stress (smaller submissive mice housed next to larger dominant mice) on fear learning and subsequent fear memory, measured as time spent “freezing“ when placed in an arena (context), HC-070 treatment reduced freezing behavior (context fear memory). Furthermore, a more substantial decrease in duration of freezing across conditioned stimulus trials was observed in mice treated with HC-070 versus controls (Fig. 2F) [59].

Marble burying by rodents is thought to share features of compulsive behaviors, encompassing anxiety; therefore, HC-070 was also evaluated in this model [59, 60]. Mice treated with vehicle control buried more marbles than those treated with HC-070 or zimelidine, a selective serotonin reuptake inhibitor (SSRI) [59]. Finally, the effect of HC-070 on antidepressant-like activity was also assessed in a mouse model using the tail suspension test and forced swim test [59]. HC-070 treatment reduced the time of immobility in the suspension test, with a similar effect size observed as for the tricyclic anti-depressant, desipramine [59]. Similar results were observed in the forced swim test, with a dose-dependent reduction in immobility in HC-070-treated mice compared with vehicle control-treated mice. Locomotor activity of HC-070-treated mice was not affected, indicating that the effects of HC-070 are not merely secondary to motor effects [59]. Elevations in glutamate levels induced by restraint stress were also attenuated in HC-070-treated rats compared with vehicle control-treated rats, suggesting that the behavioral effects observed with HC-070 may be partially attributed to reduced glutamate transmission [61]. Overall, the results from these preclinical studies suggest that acute inhibition of TRPC4/5 with HC-070 reduces anxiety and depression-like behaviors in rodent models [59].

## Pharmacokinetic and safety profile of BI 1358894

Given the strength of the preclinical data supporting the therapeutic potential for TRPC4/5 inhibition to elicit anxiolytic and antidepressant effects, BI 1358894, a small molecule inhibitor of TRPC4/5 ion channels has been developed and is currently under investigation for the treatment of MDD and post-traumatic stress disorder [62, 63]. The safety, tolerability, and pharmacokinetics of oral BI 1358894 in healthy male volunteers was evaluated in a single rising dose (SRD) Phase I study (NCT03210272/1402-001) and a separate multiple rising dose (MRD) Phase I study (NCT037549595/1402-0002) [63]. Both studies enrolled male participants, aged 18–45 years, with a BMI of 18.5–29.9 kg/m^2^. In the SRD study, BI 1358894 was administered to volunteers (*N* = 82) in a fasted or fed state to evaluate the impact of food on BI 1358894 pharmacokinetics [63]. In the MRD study (*N* = 50), a midazolam microdosing approach was applied to evaluate if BI 1358894 induces CYP3A4 after multiple dosing [63, 64].

### Pharmacokinetics

After single dosing, exposure to BI 1358894 increased in a dose-dependent manner over 5–50 mg in fasted volunteers whereas at higher doses up to 200 mg, exposure increased less than dose-proportionally [63]. Following a single 3–200 mg dose, BI 1358894 reached maximum plasma concentration (C_max_) within 1.0–5.0 h in fasted volunteers. Similarly, after multiple dosing, BI 1358894 exposure increased dose dependently but less than dose proportionally in all dose groups (10 to 200 mg). Slight accumulation occurred with repeated dosing until steady state; accumulation ratios of BI 1358894 ranged from 1.6 to 1.8 for C_max,_ and from 2.3 to 2.6 for area under the concentration–time curve over a uniform dosing interval (AUC_τ_). Volunteers reached steady state by Day 9–11, indicating an effective terminal half-life (t_1/2_) of approximately 50 h [63]. Across both studies BI 1358894 exhibited a multicompartmental decline in plasma levels with a long, slow terminal phase and a terminal t_1/2_ of 135–361 h [63]. The effect of concomitant food was tested after single dosing; compared with the fasted state, a 1.6-fold higher exposure was reported for BI 1358894 50 mg and 2.5-fold higher exposure for BI 1358894 100 mg in a high fat, high calorie fed state. Under fed conditions both area under the concentration–time curve extrapolated to infinity (AUC_0–∞_) and C_max_ were dose proportional across doses of 50–200 mg [63]. The exposure to midazolam did not change with repeated dosing of BI 1358894 for 14 days across the dose range in the MRD study, hence no clinically relevant effects of BI 1358894 on cytochrome P450 3A (CYP3A) activity were observed [63].

## Safety

In the SRD study, a higher proportion of investigator-defined drug-related adverse events (DRAEs) and treatment-related adverse events (AEs) were reported in volunteers who received BI 1358894 vs. placebo [63]. However, in general, DRAE frequency did not appear to show any dose-dependency across the SRD and MRD studies [63]. No AEs of severe intensity were reported in either study [63]. Furthermore, the incidence of AEs reported under fed conditions was similar to the incidence under fasted conditions [63]. Overall, safety findings from the SRD and MRD studies indicate that single and multiple doses of BI 1358894 were generally well-tolerated in healthy male volunteers at doses up to 200 mg [63].

### Human interventional studies

Following the SRD study and in parallel to the MRD study, two human interventional studies to demonstrate target engagement of BI 1358894 were initiated. The first study evaluated the pharmacodynamic effects of a single dose of BI 1358894 in the CCK-4 challenge model in healthy volunteers. The second study looked at the effects of a single dose of the compound on brain activation patterns while viewing pictures of faces expressing varying emotions and affective scenes, as measured by fMRI in participants with MDD.

### CCK-4 challenge study

Numerous studies have demonstrated the efficacy of various drugs to attenuate panic symptoms in the CCK-4 challenge model, including the CCK receptor antagonist CI-988, tricyclic antidepressants, SSRIs, and benzodiazepines [57, 58, 65–67]. From animal studies, it is known that CCK-4 mediates neuronal activation via the TRPC ion channels [59], with intracellular blockade of TRPC5 significantly reducing CCK-induced increases in action potential firing frequency [68]. Building upon the promising effects of HC-070 in preclinical studies, the CCK-4 challenge model was used as a translational task in healthy volunteers. Therefore, the effect of the TRPC4/5 inhibitor BI 1358894 in the CCK-4 challenge model was evaluated [62]. In the study, twenty male CCK-4-sensitive healthy volunteers were examined in a Phase I, randomized, double-blind, two-way cross-over, single administration, placebo-controlled trial. Determined following a CCK-4 IV bolus injection at screening, CCK-4 sensitivity was defined as a sudden onset of anxiety and panic along with the presence of at least four symptoms in the Panic Symptom Scale (PSS) with either a score of ≥ 2 on PSS item 15 (anxiety and fear for apprehension) or achievement of a total PSS sum intensity score > 20 [62]. BI 1358894 100 mg or placebo were administered 5 h prior to the administration of CCK-4. BI 1358894 dose was selected based on the expected pharmacodynamic effects of a single dose of BI 1358894 and the data obtained in the SRD trial [63]. The maximum change from baseline of the PSS sum intensity score after CCK-4 injection served as the primary endpoint. In addition to safety and tolerability measures of BI 1358894, further endpoints of the study were changes in the emotional faces visual analog score (EVAS), the Spielberger State-Trait Anxiety Inventory (STAI), plasma adrenocorticotropic hormone (ACTH), and serum cortisol values. For details regarding study design and the results, please see Goettel M, et al. 2023 [62].

**Fig. 3:**
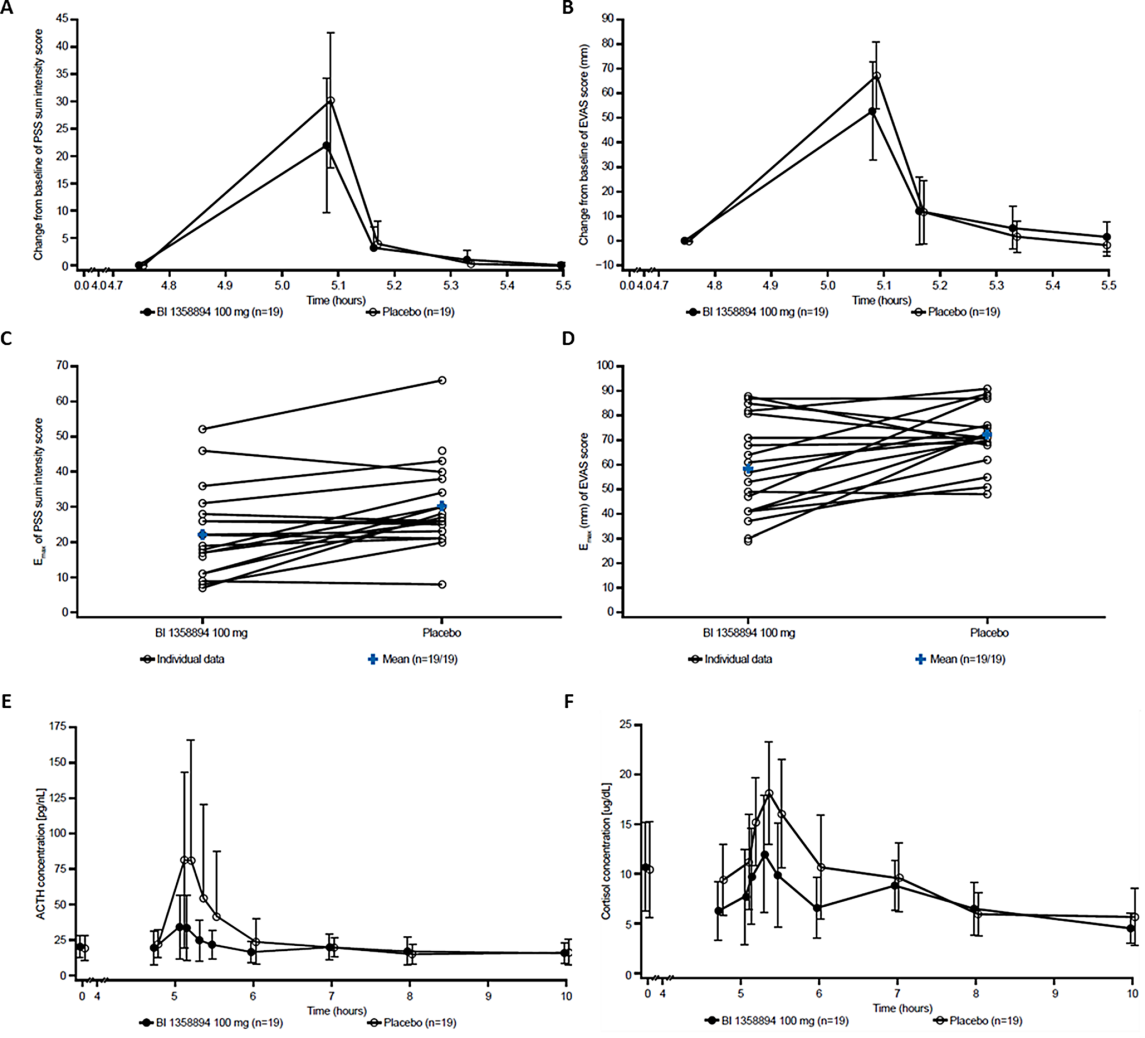
**In the CCK-4 challenge study, BI 1358894 reduced psychological and physiological responses.** Mean effect–time profiles of **(A)** PSS change from baseline (± SD); **(B)** EVAS change from baseline (± SD). As well as intra-individual and mean comparisons of **(C)** maximum PSS sum intensity score and **(D)** maximum EVAS scores. Mean effect–time profiles of **(E)** ACTH and **(F)** cortisol (± SD) in blood during the CCK-4 challenge after a single administration of BI 1358894 100 mg or placebo are presented. A single dose of BI 1358894 or placebo were administered 5 h prior to CCK-4 challenge. ACTH, adrenocorticotropic hormone; CCK-4, cholecystokinin-tetrapeptide; E_max_, maximum PSS/EVAS score or change from baseline; EVAS, emotional faces visual analog scale; PSS, Panic Symptom Scale; SD, standard deviation. Reproduced from Goettel M, et al. *CNS Drugs*. 2023;37(12):1099–110 with permission from Springer Nature.

Figure 3 summarizes the results of the CCK-4 study. In healthy volunteers treated with BI 1358894 compared with placebo, the adjusted mean maximum change from baseline in PSS sum intensity score was 24.4% lower and the adjusted mean maximum change from baseline in EVAS was reduced by 19.2% (Fig. 3A–D). Relative to placebo, BI 1358894 reduced CCK-4-induced mean maximum plasma ACTH and serum cortisol values by 58.6% and 27.3%, respectively (Fig. 3E and F). The STAI total score before CCK-4 injection was comparable in both groups (data not shown). DRAEs were reported for 65.0% of the subjects administered with BI 1358894. However, no serious or severe AEs, AEs of special interest, or AEs leading to discontinuation of trial medication, or deaths were reported. In conclusion, the study demonstrated that BI 1358894 reduces psychological (as measured by PSS and subjective EVAS) as well as physiological (as measured by objectively measured stress biomarkers including maximum plasma ACTH and serum cortisol) responses to CCK-4 compared with placebo. The study also showed that BI 1358894 had a positive safety profile and was well tolerated after a single oral dose.

## fMRI study

**Fig. 4:**
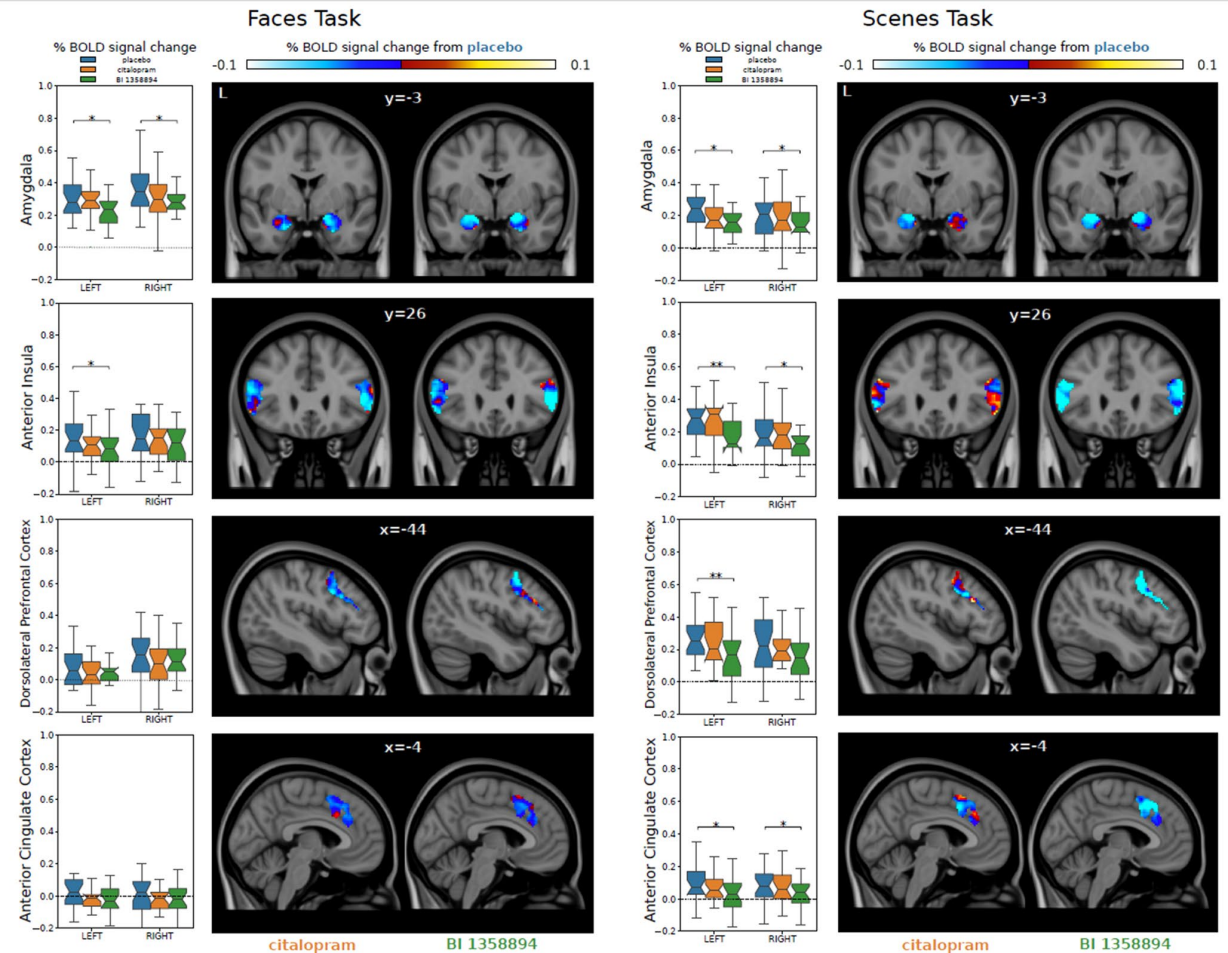
**In the fMRI study, BI 1358894 significantly reduced activation in several brain regions involved in emotional processing.** Effects of placebo, citalopram, and BI 1358894 on mean % BOLD signal change shown in ROIs for the faces and scenes task, respectively. Box plots illustrate the median across participants of the average % BOLD signal change within each ROI, with notches indicating the 95% confidence interval of the median; the lower whisker shows quartile 1–1.5 x the interquartile range; the upper whisker shows quartile 3 + 1.5 x the interquartile range. Horizontal brackets above the box plots indicate differences in adjusted means from placebo at *p < 0.05 and **p < 0.01 (ANOVA). Brain slices illustrate the difference in % BOLD signal change between placebo and each respective compound; blue color indicates a decrease in % BOLD signal change in the compound group compared with placebo, red color indicates an increase in % BOLD signal change in the compound group compared with placebo. ANOVA, analysis of variance; BOLD, blood oxygenation level dependent; fMRI, functional magnetic resonance imaging; ROI, regions of interest. Reproduced from Grimm S, et al. *Eur Neuropsychopharmacol.* 2022;65:44–5 under the terms of the Creative Commons CC-BY License (http://creativecommons.org/licenses/by/4.0/).

In a second study in 73 patients with MDD, fMRI was used to investigate the acute effects of BI 1358894 on corticolimbic neural responses during negative emotional processing [69]. In a single-dose, randomized, placebo-controlled design, BI 1358894 was compared with placebo to determine whether it can modulate neural responses to negative emotional stimuli. Citalopram was compared with placebo to ensure that the study design was sufficiently sensitive to detect the effects of an antidepressant known to induce effects in the fMRI paradigm, and therefore served as a positive control. Inclusion criteria for the study were a diagnosis of MDD according to the Diagnostic and Statistical Manual of Mental Disorders, Fifth Edition (DSM-5), with a total score between > 7 and < 26 on the Montgomery-Åsberg Depression Rating Scale (MADRS) [70], and aged between 18 and 45 years. Treatment allocation was 1:1:1 and randomization was stratified by the severity of depression (MADRS ≥ 20 and < 20). Exclusion criteria were any other major mental disorder, intake of prescribed medication (including antidepressants), history of relevant neurological diseases, migraine headaches, relevant medical condition, MRI exclusion criteria, and pregnancy. Participants were randomized to receive a single oral dose of either BI 1358894 100 mg, citalopram 20 mg, or a matched placebo tablet. Participants were administered BI 1358894 or placebo, then citalopram or placebo 3 h later. Scanning commenced after a further 3 h (i.e., 6 h after BI 1358894 administration). The citalopram dose selected for this trial reflects standard clinical dosage and has been used previously in trials with a similar design [23, 25]. The BI 1358894 dose was selected based on the expected pharmacodynamic effects of a single dose and the data obtained in the SRD trial [63]. Patients completed two fMRI tasks probing emotional processing [71, 72]. First, an emotional face viewing task in which they were shown negative emotional faces (fear, disgust, sadness) from the Warsaw Set of Emotional Facial Expression Pictures (WSEFEP) [73]. Second, participants were shown negative scenes from the Open Affective Standardized Image Set (OASIS) picture set [74]. Brain images were acquired using a 3 Tesla MRI scanner (PRISMA, Siemens Medical Systems, Erlangen, Germany) with a 64-channel head coil and a T2∗-weighted gradient echo-planar imaging sequence (TR = 2 s, TE = 30 ms, flip angle = 80°, voxel size = 3 × 3 × 3 mm, matrix 64 × 64, 36 slices, FOV = 192 × 192 × 143 mm, GRAPPA acceleration factor 2). All image preprocessing and first-level analyses were carried out using FEAT (fMRI Expert Analysis Tool) Version 6.0, part of fMRIB’s Software Library (FSL, www.fmrib.ox.ac.uk/fsl) [75]. Main analyses focused on the average % blood oxygenation level dependent (BOLD) signal change within prespecified regions of interest (ROIs; bilateral amygdala, anterior insula, DLPFC, and ACC). For each region, separate analysis of variance (ANOVA) models were computed for the comparison of treatments (BI 1358894 or citalopram) versus placebo. The adjusted treatment differences in the percent BOLD signal changes in the faces task showed that BI 1358894 induced signal reduction in bilateral amygdala and left anterior insula. In the scenes task, BI 1358894 produced significant signal reduction in bilateral amygdala, anterior insula, ACC, and left DLPFC. Citalopram failed to induce any significant reductions in BOLD signal in both tasks (Fig. 4) [69]. These fMRI findings thereby demonstrated that BI 1358894 significantly reduces activation in several brain regions involved in emotional processing [69].

## Discussion

Preclinical data demonstrated that treatment with a TRPC4/5 antagonist attenuates the anxiogenic effect of CCK-4 in the elevated plus maze and recapitulates the phenotype observed in both null TRPC4 and TRPC5 mice. Anxiolytic and antidepressant-like effects of a TRPC4/5 antagonist were also observed in pharmacological in vivo tests including marble burying, tail suspension, forced swim and in fear memory induced by chronic social stress [59]. Furthermore, brain slices showed that application of the TRPC4/5 antagonist substantially reduced the frequency of spontaneous excitatory postsynaptic currents in the basolateral amygdala stimulated by the panic-inducing peptide CCK-4 [59]. These results provided strong evidence that acute inhibition of TRPC4 and TRPC5 reduces behavior associated with anxiety and depression in mouse models. Findings from subsequent interventional studies in healthy volunteers and patients with MDD showed that the TRPC inhibitor BI 1358894 reduced psychological and physiological responses on CCK-4-induced anxiety/panic-like symptoms and had a broad effect on corticolimbic NV systems, confirming preclinical findings. Furthermore, BI 1358894 also had a positive safety profile and was well tolerated after a single oral dose [29, 59, 62, 69]. Previous studies in patients suggest that altered reactivity in corticolimbic NV systems, as conceptualized in the RDoC, is a central feature of symptoms associated with negative emotional processing such as anxiety, rumination, and depressed mood [12, 13, 17, 19, 20]. While TRPC channels are widely expressed throughout the brain, their transcript levels are particularly high in the frontal cortex and amygdala [30] and it has been hypothesized that TRPC inhibition may exert behavioral effects on anxiety and depression in mouse models by reducing excitability of the amygdala [59]. Previous clinical studies showed that an overactive amygdala is a major contributor to attentional bias to negative stimuli, pessimistic thoughts, and anxiety [12, 19]. Amygdala hyperactivity in response to emotional stimuli may be a neurobiological feature specific to MDD with anxious distress compared with MDD without anxious distress [21]. The effect of BI 1358894 on CCK-4-induced anxiety/panic-like symptoms in healthy volunteers supports a role for amygdala TRPC4, TRPC5, and CCK-4 activity in anxiety-like behaviors. In MDD patients, BI 1358894 affected further corticolimbic brain regions and reduced activity of the anterior insula, an area considered essential for the integration of interoceptive information and emotional experience [76] and in generating appropriate behavioral responses to salient external or internal stimuli [77]. Increased insula activity in MDD has been related to abnormal emotional processing, with preferential processing of negative information [78, 79]. BI 1358894 furthermore elicited a broad and robust circuit engagement by means of attenuated activity in ACC and DLPFC. Results of previous fMRI studies investigating these regions in healthy volunteers via acute pharmacological modulation using citalopram were complemented by findings in patients with MDD, in which prolonged administration of citalopram over several weeks led to decreased reactivity to negative emotional stimuli in the amygdala and insula, and increased regulatory responses in DLPFC [80–82]. Accordingly, it was speculated that through normalization of corticolimbic circuits associated with the NV symptom domain, antidepressants may eventually improve patients’ abnormalities in emotional reactivity and deficits in emotional regulation, so as to reduce depressed mood and negative emotional bias [16, 20]. TRPC4 and TRPC5 inhibition by BI 1358894 is a mechanism of action that has the potential to impact multiple transmitter systems. The channels are downstream of numerous signaling pathways including many GPCRs that signal via G-alpha q as well as Gi and Go [41, 83]. In addition, activity of TRPC5 homomultimers is strongly potentiated by calcium, linking the activity of the channel to several other signaling events [84]. Our findings of reduced psychological and physiological responses on CCK-4-induced anxiety/panic-like symptoms and of a broad effect of BI 1358894 on corticolimbic NV systems indicate a critical role for TRPC4 and TRPC5 in emotional processing. It remains an open question though, if the biological signatures of TRPC4/5 inhibition reported here translate into clinical efficacy and indicate a potential role for TRPC4/5 inhibitors in more effective treatment of internalizing disorders, such as MDD and anxiety disorders.

## Summary

In summary, TRPC inhibition is distinct from current therapeutics. The broad circuit modulation through BI 1358894 supports the hypothesis that it may have the potential to provide relief to patients who do not respond to current therapies, which should be further investigated in studies in patients with NV system disorder, like MDD and PTSD.

## Data Availability

To ensure independent interpretation of clinical study results and enable authors to fulfil their role and obligations under the ICMJE criteria, Boehringer Ingelheim grants all external authors access to relevant clinical study data. In adherence with the Boehringer Ingelheim Policy on Transparency and Publication of Clinical Study Data, scientific and medical researchers can request access to clinical study data, typically, one year after the approval has been granted by major Regulatory Authorities or after termination of the development program. Researchers should use the https://vivli.org/ link to request access to study data and visit https://www.mystudywindow.com/msw/datasharing for further information.
